# ArfA recognizes the lack of mRNA in the mRNA channel after RF2 binding for ribosome rescue

**DOI:** 10.1093/nar/gku1069

**Published:** 2014-10-29

**Authors:** Daisuke Kurita, Yuhei Chadani, Akira Muto, Tatsuhiko Abo, Hyouta Himeno

**Affiliations:** 1 Department of Biochemistry and Molecular Biology, Faculty of Agriculture and Life Science, Hirosaki University, Hirosaki 036–8561, Japan; 2 Graduate School of Natural Science and Technology, Okayama University, Okayama 700–8530, Japan; 3 Department of Biology, Faculty of Science, Okayama University, Okayama 700–8530, Japan

## Abstract

Although *trans*-translation mediated by tmRNA-SmpB has long been known as the sole system to relieve bacterial stalled ribosomes, ArfA has recently been identified as an alternative factor for ribosome rescue in *Escherichia coli*. This process requires hydrolysis of nascent peptidyl-tRNA by RF2, which usually acts as a stop codon-specific peptide release factor. It poses a fascinating question of how ArfA and RF2 recognize and rescue the stalled ribosome. Here, we mapped the location of ArfA in the stalled ribosome by directed hydroxyl radical probing. It revealed an ArfA-binding site around the neck region of the 30S subunit in which the N- and C-terminal regions of ArfA are close to the decoding center and the mRNA entry channel, respectively. ArfA and RF2 sequentially enter the ribosome stalled in either the middle or 3′ end of mRNA, whereas RF2 induces a productive conformational change of ArfA only when ribosome is stalled at the 3′ end of mRNA. On the basis of these results, we propose that ArfA functions as the sensor to recognize the target ribosome after RF2 binding.

## INTRODUCTION

Protein synthesis is terminated when a stop codon appears in the decoding center. In bacteria, the class I release factors, RF1 and RF2, enter the A-site in a stop codon-dependent fashion to perform polypeptide release from the peptidyl-tRNA in the P-site, which is catalyzed by a universally conserved Gly-Gly-Gln (GGQ) motif of release factor. When a truncated mRNA lacking a stop codon (non-stop mRNA) is translated, the ribosome stalls at the 3′ end of mRNA, as neither RF1 nor RF2 functions. Since accumulation of excess stalled ribosomes is deleterious to the cells, each bacterium should possess any system(s) to rescue them (1). *Trans*-translation mediated by tmRNA and SmpB has been considered as the bacterial standard system, considering its universal distribution among the bacterial domain (2). In this system, tmRNA enters the ribosome stalled at the 3′ end of non-stop mRNA and receives the nascent polypeptide from the peptidyl-tRNA. And then, translation is switched from non-stop mRNA to the open reading frame on tmRNA, which encodes a tag-peptide preferentially recognized by cellular ATP-dependent degradation systems as represented by the SspB-ClpXP degradation system. Thus, it facilitates ribosome recycling for a new round of translation and avoids the resulting tagged polypeptides from accumulation in the cell. Although *trans*-translation is essential for the viability of some bacteria, such as *Neisseria gonorrhoeae*, *Helicobacter pylori* and *Shigella flexneri* (3–5), it is dispensable for many other bacteria including *Escherichia coli*. It raises the possibility of the existence of alternative rescue system(s) for the stalled ribosome.

Recently, ArfA (YhdL) has been identified as a factor required for *E. coli* lacking *ssrA* (the gene for tmRNA) (6,7). Although *arfA* (the gene for ArfA) is not essential, disruptions of *ssrA* and *arfA* result in a synthetically lethal phynotype. The *arfA* mRNA is usually cleaved by RNase III followed by *trans*-translation-dependent degradation (8–10), and consequently functional ArfA protein is expressed from this truncated mRNA only when *trans*-translation does not sufficiently work, suggesting that the ArfA-dependent rescue system acts as a backup system for *trans*-translation in the cell. ArfA does not contain the GGQ motif, which is essential for release of polypeptide by class I release factors, leading to another finding that ArfA requires RF2, but not RF1, for peptidyl-tRNA hydrolysis (11,12). While RF2 usually recognizes the UAA or UGA stop codon to terminate translation, the stop codon is missing in non-stop mRNA. This deepens the mystery of the molecular mechanism of the ArfA-dependent rescue system.

In the present work, we studied the sites and modes of binding of ArfA to the ribosome by directed hydroxyl radical probing. It revealed an ArfA-binding site on the ribosome; the N-terminal region of ArfA is located around the decoding center, while the C-terminal region is located along the mRNA entry channel downstream of the A-site. We also found that cleavage patterns are considerably affected by the addition of RF2, suggesting that ArfA undergoes a significant conformational change by RF2. Using fluorescence spectroscopy, we determined the dissociation constant between ArfA and either 70S ribosome, ribosomal subunits or RF2. It revealed that ArfA binds to the 70S ribosome or 50S subunit with similar affinity and much less efficiently to the 30S subunit or RF2. Intriguingly, ArfA and RF2 bind to the ribosome regardless of the length of the 3′ extension of mRNA, although they catalyze peptidyl-tRNA hydrolysis only in the ribosome stalled near the 3′ end of mRNA. Based on these results, we propose a molecular mechanism underlying the ArfA-dependent ribosome rescue system.

## MATERIALS AND METHODS

### Preparation of ArfA, RF1, RF2, ribosome and charged tRNAs

Mutations were introduced into a plasmid for overproduction of His-tagged ArfA-ΔC17 from *E. coli* (9). ArfA derivatives, RF1 and RF2 were prepared as previously described (11). 70S ribosomes were prepared from *E. coli* W3110 as described (13). tRNA^fMet^ was purchased from Sigma-Aldrich Co. *N*-formyl-[^14^C]Met-tRNA^fMet^ was prepared as described (14).

### Peptidyl-tRNA hydrolysis assay

The stalled ribosome was formed by incubating 20 pmol ribosomes, 100 pmol mRNA and 40 pmol *N*-formyl-[^14^C]Met-tRNA^fMet^ in 40 μl of buffer A for 10 min at 37°C. Buffer A contains 80 mM Tris-HCl (pH 7.8), 7 mM MgCl_2_, 150 mM NH_4_Cl, 2.5 mM dithiothreitol and 2 mM spermidine. After incubation, stalled ribosome mixture was incubated with 10 μl of 50 pmol ArfA and 50 pmol RF2-B in buffer A. At specified times, reaction mixture was quenched in 150 μl of 0.1 M HCl. Hydrolyzed *N*-formyl-[^14^C]Met was extracted with 300 μl of ethyl acetate and 200 μl of the ethyl acetate layer was spotted onto glass fiber filter (Advantec). Radioactivity on the filter was measured by a liquid scintillation counter.

### Conjugation of Fe(II)-BABE to ArfA

Three nmol of each ArfA derivative was incubated with 30 nmol Fe(II)-bromoacetamidobenzyl-EDTA (BABE) in 100 μl of solution containing 50 mM MOPS (pH 8.2), 100 mM NaCl, 1 mM ethylenediaminetetraacetic acid (EDTA) and 5% glycerol at 37°C for 1 h. Free Fe(II)-BABE was removed by gel filtration. A mock modification was also performed on wild type ArfA-ΔC17, which has no cysteine residue, as the control.

### Formation of the complex of ArfA and ribosome

70S ribosomes (20 pmol) were incubated with a synthetic mRNA (100 pmol) and tRNA^fMet^ (100 pmol) at 37°C for 10 min. They were subsequently incubated with Fe(II)-tethered ArfA (300 pmol) and RF2-B (0 or 50 pmol) in 50 μl of 50 mM MOPS (pH 8.0), 120 mM NaCl, 0.1 mM EDTA, 10 mM MgCl_2_ and 10% glycerol at 37°C for 10 min.

### Directed hydroxyl radical probing

Ribosome in complex with Fe(II) tethered ArfA was probed by initiating hydroxyl radical formation with 6 μl of 250 mM ascorbic acid and 6 μl of 1.25% H_2_O_2_. The reaction mixtures were incubated on ice for 10 min and quenched with 100 mM thiourea. RNA was prepared by phenol extraction and ethanol precipitation. Reverse transcriptase reaction was carried out in a 12 μl reaction mixture containing 50 mM Tris-HCl (pH 8.3), 75 mM KCl, 3 mM MgCl_2_, 10 mM dithiothreitol, 0.5 mM each of dNTP, 1 pmol of rRNA, 1 pmol of 5′Texas Red labeled DNA primer complementary to a portion of the rRNA sequence, three units of ribonuclease inhibitor (Takara) and 18 units of reverse transcriptase (Takara). After the addition of 3 μl of stop solution containing 95% formamide, 0.5 mM EDTA and 0.1 mg/ml Fuchsin Red, the positions of cleavages were analyzed using a fluorescence DNA sequencer (Hitachi SQ-5500).

### Fluorescence polarization analysis

ArfA was labeled with 5-carboxytetramethylrhodamine (TAMRA) using the Protein Labeling Kit MF-543PX (Olympus) according to the instructions. To form the stalled ribosome, indicated amounts of 70S ribosomes, mRNA (mRNA + 0 or mRNA + 21, 100 pmol) and tRNA^fMet^ (100 pmol) were incubated in 25 μl of solution containing 80 mM Tris-HCl (pH 7.5), 7 mM MgCl_2_, 150 mM NH_4_Cl, 2.5 mM dithiothreitol, 2 mM spermidine and 0.05% Tween 20 at 37°C for 10 min. Labeled ArfA (100 fmol) in 25 μl of solution containing 80 mM Tris-HCl (pH 7.5), 7 mM MgCl_2_, 150 mM NH_4_Cl, 2.5 mM dithiothreitol, 2 mM spermidine and 0.05% Tween 20 was mixed with the ribosome mixture and incubated at 37°C for 10 min. The reaction mixtures were applied to a glass-bottomed microplate and they were set in MF20 fluorescence spectroscopy system (Olympus). The measurement of polarization was performed with excitation wavelength at 543 nm and laser power of 100 μW. Data acquisition time was 5 s per measurement.

### Ultrafiltration assay

70S ribosomes (20 pmol), mRNA (mRNA + 0 or mRNA + 21, 100 pmol) and tRNA^fMet^ (100 pmol) were incubated in 25 μl of solution containing 80 mM Tris-HCl (pH 7.5), 7 mM MgCl_2_, 150 mM NH_4_Cl, 2.5 mM dithiothreitol and 2 mM spermidine at 37°C for 10 min. ArfA (20 pmol) and RF2 (0, 1.9, 5.6, 17 and 50 pmol) in 25 μl of solution containing 80 mM Tris-HCl (pH 7.5), 7 mM MgCl_2_, 150 mM NH_4_Cl, 2.5 mM dithiothreitol and 2 mM spermidine were mixed with the ribosome mixture and incubated at 37°C for 10 min. Free RF2 and ArfA were removed by centrifugal ultrafiltration using Amicon Ultra (100 kDa). Ribosome fraction was subjected to SDS-PAGE and visualized by Oriole Fluorescent Gel Stain (Bio-Rad).

## RESULTS

### Single cysteine derivatives of ArfA

We used site-directed mutagenesis to construct single-cysteine mutants of ArfA for attaching Fe(II)-BABE probes. In previous *in vivo* studies, functional ArfA protein is synthesized from *arfA* mRNA cleaved by RNase III as the C-terminally truncated product (8,9). Therefore, we used ArfA-ΔC17 construct, which lacks the C-terminal 17 amino acids, as the wild-type ArfA. The active form of wild type ArfA contains no naturally occurring cysteine residue, and we introduced a single cysteine-residue at each of 34 different positions of wild-type ArfA. The mutant ArfA proteins were overexpressed, purified by Ni-NTA agarose chromatography, and Fe(II)-BABE was attached to the cysteine residue in each ArfA variant for directed hydroxyl radical probing. This approach uses locally generated hydroxyl radicals to cleave rRNA in the vicinity of Fe(II) tethered to the unique cysteine residue introduced into ArfA.

**Figure 1.**
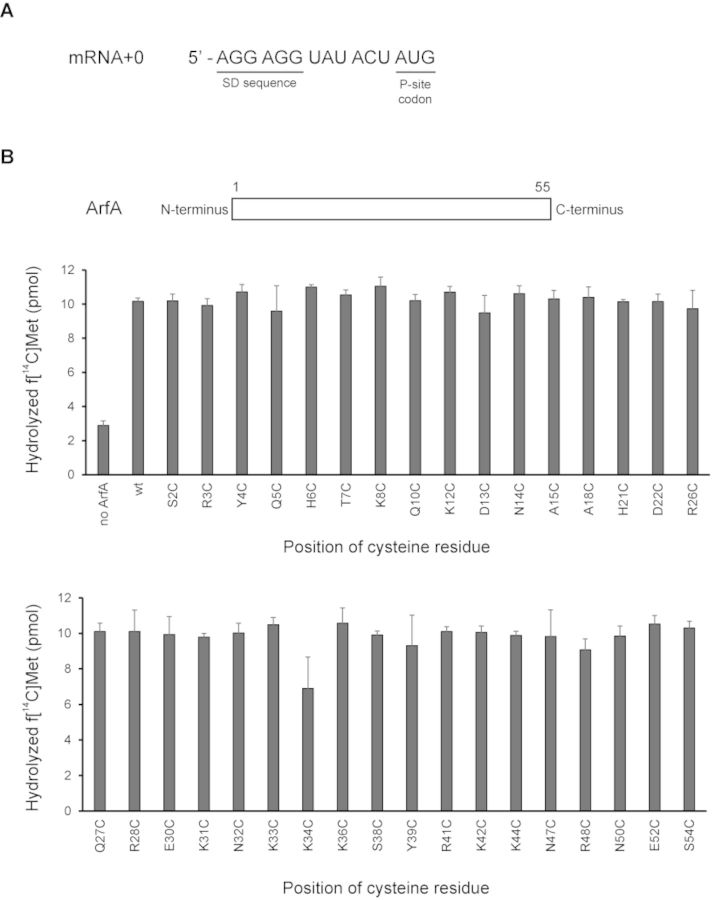
Activity of *in vitro* peptidyl-tRNA hydrolysis for ArfA variants. (**A**) mRNA sequence used in PTH assay. (**B**) Stalled ribosome with the P-site occupied by *N*-formyl-[^14^C]Met-tRNA^fMet^ and a short mRNA encompassing the SD sequence to the P-site AUG codon (mRNA + 0) was incubated with ArfA and RF2 for 10 min at 37°C. After hydrolysis, *N*-formyl-[^14^C]Met was extracted by ethyl acetate and radioactivity was measured by a liquid scintillation counter. Data are means with standard deviations and are representative of at least three independent experiments.

To assess the activities of these mutants prior to labeling with Fe(II)-BABE, we developed an *in vitro* peptidyl-tRNA hydrolysis (PTH) assay system using purified components. In this system, 70S ribosome was incubated with an mRNA encompassing the SD sequence to the P-site AUG codon (Figure 1A) and *N*-formyl-[^14^C]Met-tRNA^fMet^ instead of peptidyl-tRNA^fMet^ to form the stalled ribosome. Then, this stalled ribosome with the P-site occupied by *N*-formyl-[^14^C]Met-tRNA^fMet^ and the vacant A-site was incubated with ArfA and RF2. It has been reported that overexpressed RF2 from *E. coli* K12 strain (RF2-K) has a lower activity of peptide release than that from B strain (RF2-B) in terms of termination of translation (15) and ArfA-dependent peptidyl-tRNA hydrolysis (11). Therefore, we prepared RF2-B as previously described (11). After hydrolysis of *N*-formyl-[^14^C]Met-tRNA^fMet^, released *N*-formyl-[^14^C]Met was extracted by ethyl acetate and radioactivity was measured by a liquid scintillation counter. We confirmed that most ArfA mutants have PTH activity comparable to wild-type ArfA, although K34C mutant exhibited a slight decrease (Figure 1B). The PTH activity of A18C mutant was comparable to that of wild type ArfA in our assay system, although A18T mutant has been reported to have a defect in release of nascent polypeptide from the stalled ribosome in a cell-free translation system (11,12).

### Directed hydroxyl radical probing of ArfA/70S complex

**Figure 2.**
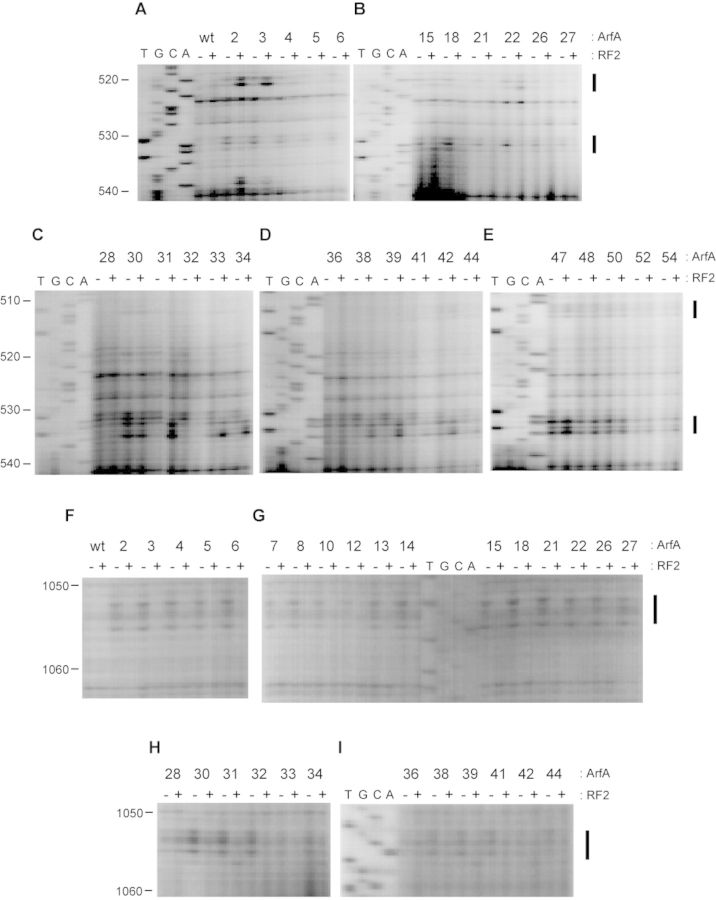

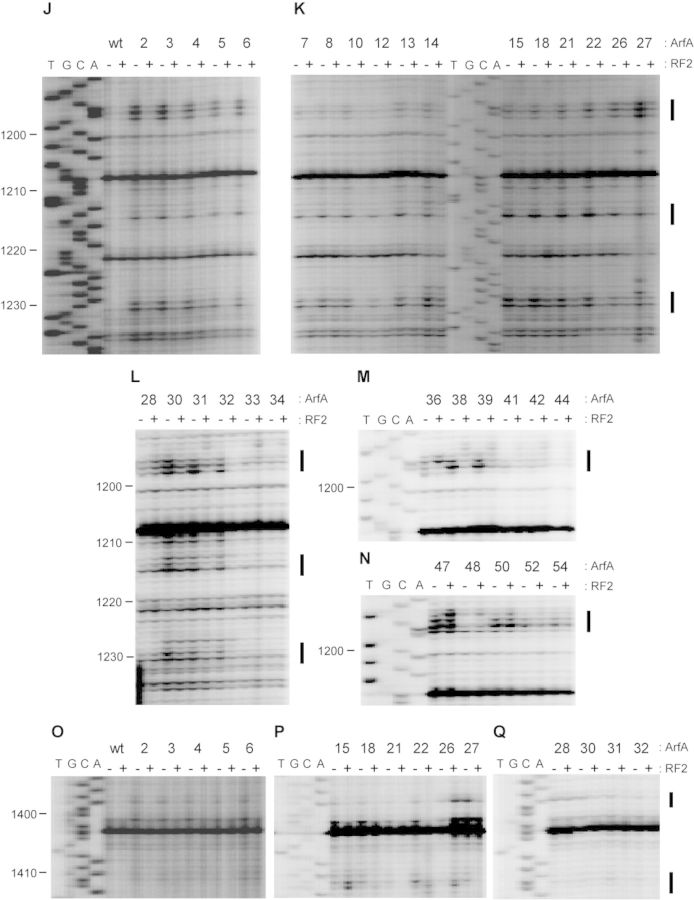
Directed hydroxyl radical probing of 16S rRNA. (**A**–**Q**) The sites of cleavages were detected by primer extension. Stalled ribosome with the P-site occupied by tRNA^fMet^ and a short mRNA (mRNA + 0, the sequence of which is shown in Figure 1A was probed with ArfA variants in the absence (−) and presence (+) of RF2. The letters A, C, G and T indicate sequencing lanes. All other lanes are from the stalled ribosomes probed with wild-type ArfA and ArfA mutants each having a single cysteine residue at the indicated position. The bands of interest are designated by bars.

Fe(II)-ArfA was mixed with stalled ribosome complex containing 70S ribosome, an mRNA encompassing the SD sequence to the P-site AUG codon (the sequence is shown in Figure 1A) and tRNA^fMet^. Then, cleavages of 23S rRNA and 16S rRNA by hydroxyl radicals generated from the tethered Fe(II) were detected by primer extension. It has been reported that ArfA preferably binds the 50S subunit rather than the 30S subunit (6). However, cleavage sites detected were all localized in 16S rRNA (Figure 2A–Q). Fe(II) tethered to positions 2, 3, 4 and 5 in the N-terminal region of ArfA cleaved nucleotides in 16S rRNA helix 18 (531–532), which flank on the decoding center (Figure 2A and B). Positions 30, 32, 38 and 39 in the C-terminal region of ArfA also cleaved the same site (Figure 2C and D). Positions 47, 48 and 50 in the C-terminal region of ArfA cleaved nucleotides in 16S rRNA helix 18 (511–513 and 533–535) between the decoding center and the mRNA entry channel (Figure 2E). 16S rRNA helices 30 and 32 (1053–1055, 1195–1197, 1215 and 1230–1231) near the decoding center are cleaved by some N-terminal mutants (positions 2 to 32, Figure 2F–N). Positions 38, 39, 47 and 50 in the C-terminal region of ArfA also cleaved nucleotides in helix 34 (1195–1197, Figure 2M and N). Positions 22 and 27 in the N-terminal region of ArfA cleaved nucleotides in helix 44 (1398 and 1411–1412, Figure 2O–Q). These cleavages provide information about a set of proximity relationships between specific residues of ArfA and rRNA residues of the ribosome.

### Effect of RF2 binding on probing

Since ArfA requires RF2 for peptidyl-tRNA hydrolysis, we tested the effect of RF2 binding to the ribosome on the pattern of directed hydroxyl radical probing. Interestingly, the cleavage pattern was significantly changed when RF2 was added (Figure 2A–Q and Table 1). Cleavages in helix 18 (520–521) by Fe(II) tethered to positions 2 and 3 in the N-terminal region of ArfA became detectable only after the addition of RF2 (Figure 2A). Positions 33, 34, 38 and 39 in the first half of the C-terminal region of ArfA more strongly cleaved nucleotides in helix 18 (533–535) in the presence of RF2 (Figure 2C and D). These nucleotides were also cleaved from Fe(II) tethered to position 47, 48 or 50 in the latter half of the C-terminal region of ArfA regardless of the presence or absence of RF2 (Figure 2E). On the other hand, cleavages in helix 18 (531–532) from positions 38 and 39 became less intensified by the addition of RF2 (Figure 2D), and cleavages in helices 30, 32 and 34 (1053–1055, 1195–1197, 1215 and 1230–1231) by N-terminal mutants disappeared by the addition of RF2 (Figure 2H and L). Taken together, RF2 binding significantly changes the mode of interaction of ArfA to the stalled ribosome, especially in the N-terminal region (2–27) and the first half of the C-terminal region (28–39) of ArfA rather than the latter half (47–50).

**Table 1.** Sites of cleavage by directed hydroxyl radical probing.

Site of cleavage	Position of cysteine residue introduced			
	mRNA + 0		mRNA + 21	
	−RF2	+RF2	−RF2	+RF2
511–513	30, 47–50	30,47–50	30	30
520–521		2, 3, 22		
531–532	2–6, 18, 22, 30–42	30	30	30
533–535	30, 47–50	30–34, 38, 39, 42, 47–50	30	30
1053–1055	2–32, 42	28	18, 42	18, 42
1195–1197	2–32, 36–39, 47, 50			
1215	2–32			
1230–1231	2–32			
1398	2–6, 15, 18, 22, 26–30	26–30		
1411–1412	6, 15, 18, 22, 27	15, 18, 22, 26	18	18

### Modeling the ArfA-70S ribosome interaction

**Figure 3.**
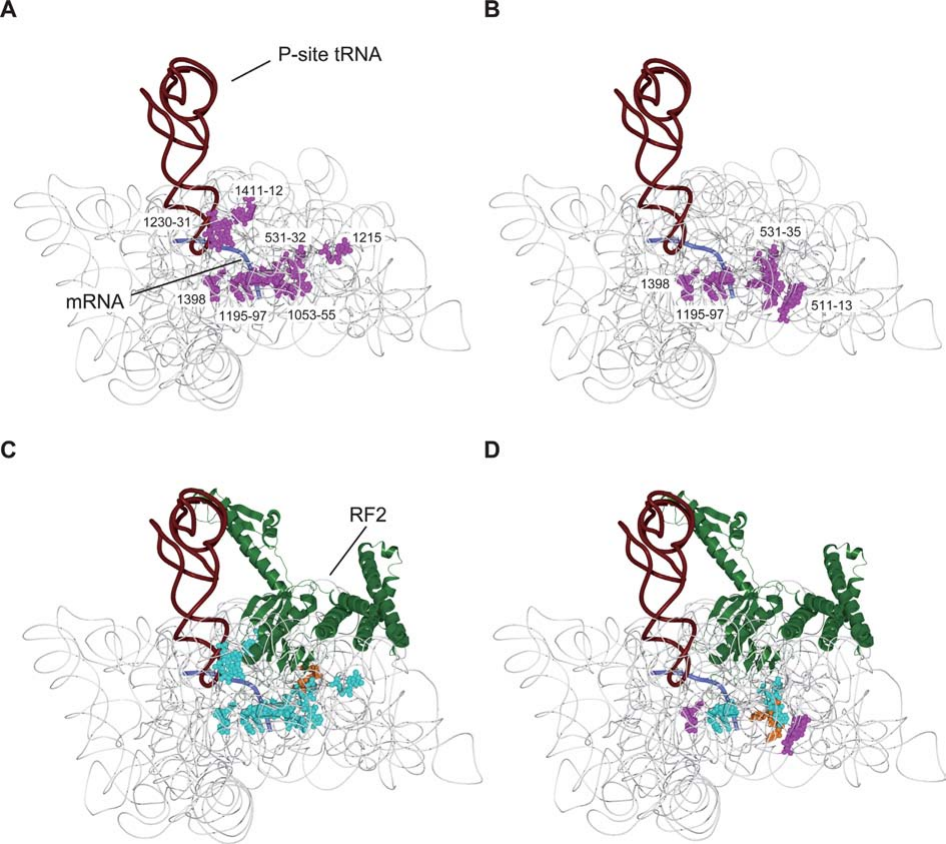
Mapping of cleavage sites in the 70S ribosome. Cleavage sites by hydroxyl radicals mapped onto the crystal structure of *Thermus thermophilus* 70S-RF2 complex (PDB 3F1G). (**A** and **B**) Cleavage sites in 16S rRNA from the (**A**) N-terminal and (**B**) C-terminal mutants of ArfA in the absence of RF2 are designated as balls (magenta). Ribosomal proteins and large subunit rRNAs are not shown for clarity. (**C** and **D**) Cleavage sites in 16S rRNA from the (**C**) N-terminal and (**D**) C-terminal mutants of ArfA in the presence of RF2 are designated as balls. Cleavages, which were weakened, strengthened and unchanged by RF2, are colored light blue, orange and magenta, respectively. Ribosomal proteins and large subunit rRNAs are not shown for clarity.

We used the probing data to model the position of ArfA in a crystal structure of the 70S ribosome with RF2 (16), although with no structural data of ArfA available. Cleavages were observed predominantly in the neck region of the 30S subunit from 30 Fe(II) tethering sites on ArfA in the absence (Figure 3A and B) and presence (Figure 3C and D) of RF2. There are two prominent regions: (i) the region around the decoding center was mainly cleaved from Fe(II) tethered to the N-terminal residues (Figure 3A and C) and (ii) the region around the mRNA entry channel was cleaved from Fe(II) tethered to some C-terminal residues (Figure 3B and D). As shown in Figure 3C and D, some cleavages were modulated by the addition of RF2. Although most cleavages from N-terminal residues were weakened or disappeared in the presence of RF2, the region 520–521 in 16S rRNA helix 18, which flanks on the decoding center, became cleaved from Fe(II) tethered to position 2 or 3 of ArfA only after the addition of RF2. On the other hand, nucleotides (533–535) that are located inside the mRNA entry channel became cleaved from Fe(II) tethered to position 33, 34, 38 or 39 in the C-terminal region of ArfA after the addition of RF2.

### ArfA binds to 70S ribosome but not RF2 *in vitro*

**Figure 4.**
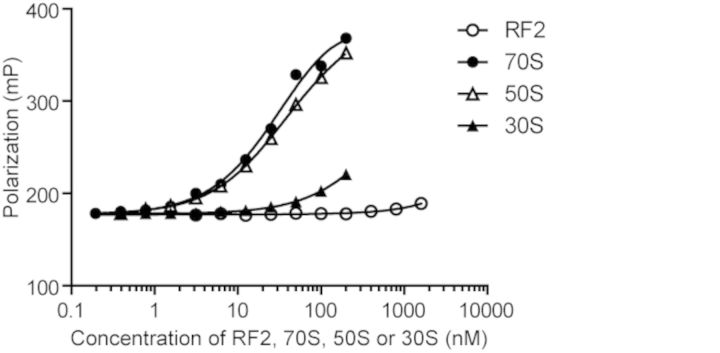
Fluorescence polarization binding analysis of the ArfA to either RF2, 70S ribosome, 50S or 30S subunit. ArfA was labeled with 5-TAMRA. Polarization was plotted against concentration of factors. Measurement was repeated five times and the means are presented.

It has been reported that ArfA preferentially co-sediments with the 50S subunit (6 ). In order to determine the dissociation constant between ArfA and either 70S ribosome, 50S or 30S subunit, we performed fluorescence polarization binding assay. ArfA labeled with 5-TAMRA (5-Carboxytetramethylrhodamine) was incubated with various concentrations of 70S ribosome, 50S or 30S subunit. As shown in Figure 4, the level of polarization, which reflects the degree of complex formation, increased with increasing concentration of the 70S ribosome or the 50S subunit. The dissociation constants between ArfA and the 70S ribosome and between ArfA and the 50S subunit were 36 and 34 nM, respectively. In contrast, we were only able to detect much lower affinity between ArfA and the 30S subunit.

In previous pull-down experiments, there is no evidence that ArfA interacts with RF2 (6,11). To measure the affinity of ArfA to RF2, labeled ArfA was incubated with various concentrations of RF2 (Figure 4). Polarization was not affected even by high concentration of RF2, indicating that ArfA does not substantially form a complex with RF2 prior to ribosome binding. This confirms that ArfA and RF2 bind to the ribosome independently rather than in complex with each other.

### ArfA and RF2 bind to the stalled ribosome regardless of the length of 3′ extension of mRNA

**Figure 5.**
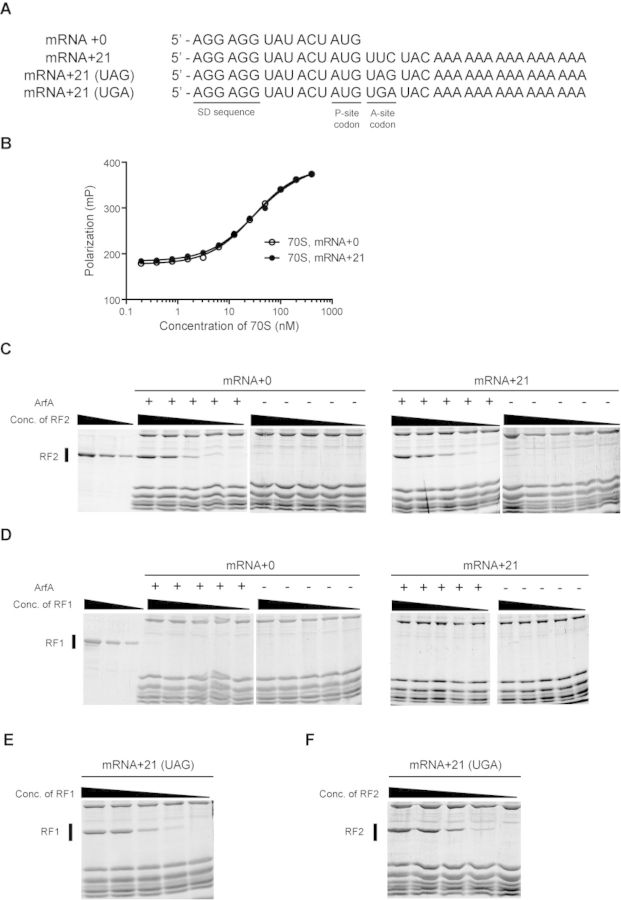
Effect of the length of 3′ extension of mRNA to ArfA and RF2 binding. (**A**) mRNA sequences used in ribosome-binding assay. (**B**) Fluorescence polarization binding analysis of 5-TAMRA-labeled ArfA to the ribosome with the P-site occupied tRNA^fMet^ and either mRNA + 0 or mRNA + 21. Polarization was plotted against concentration of ribosomes. Measurement was repeated five times. (**C** and **D**) Ribosome-binding analysis of (**C**) RF2 or (**D**) RF1 to the ribosome with ArfA, tRNA^fMet^ occupying the P-site and either mRNA + 0 or mRNA + 21. After removal of ribosome-unbound RF2 or RF1 by centrifugal ultrafiltration, ribosome fraction was subjected to SDS-PAGE. (**E** and **F**) Ribosome binding of (**E**) RF1 or (**F**) RF2 to the ribosome with tRNA^fMet^ at the P-site and mRNA + 21 containing UAG or UGA codon at the A-site.

It has been reported that the efficiency of the ArfA-dependent ribosome rescue decreases with an increase in mRNA length (12). ArfA binds around the decoding center and the mRNA entry channel, raising the possibility that ArfA competes with the 3′ extension of mRNA for the same binding site. To test the effect of the 3′ extension of mRNA on the ribosome binding of ArfA, we performed the fluorescence polarization binding assay. We used two kinds of mRNAs having 0 and 21 nucleotides extending from the P-site in consideration of the span of the downstream mRNA path from the P-site (Figure 5A). Ribosome was incubated with tRNA^fMet^ and an mRNA having a Shine-Dalgarno (SD) sequence, an AUG start codon and 0 or 21 nucleotides of 3′ extension (mRNA + 0 or mRNA + 21) to produce a stalled ribosome as previously reported (17). This stalled ribosome was incubated with 5-TAMRA-labeled ArfA. Contrary to expectation, we found that ArfA binds to the ribosome regardless of the length of the 3′ extension of mRNA (Figure 5B).

Next, we examined the effect of the 3′ extension of mRNA on ribosome binding of RF2. Ribosome stalled on either mRNA + 0 or mRNA + 21 was incubated with ArfA and various concentrations of RF2. After incubation, unbound RF2 (and ArfA) was washed out by centrifugal ultrafiltration with a 100-kDa molecular weight cut-off. Then the ribosome fraction was subjected to SDS-PAGE. We found that RF2 binds to the ribosome regardless of the length of the 3′ extension of mRNA in an ArfA-dependent manner (Figure 5C). In contrast, RF1 did not bind to the ribosome in either the presence or absence of ArfA (Figure 5D), in agreement with a previous study (12). We confirmed that RF1 and RF2 bind to the ribosome depending on the corresponding stop codons, UAG and UGA, respectively (Figure 5E and F).

**Figure 6.**
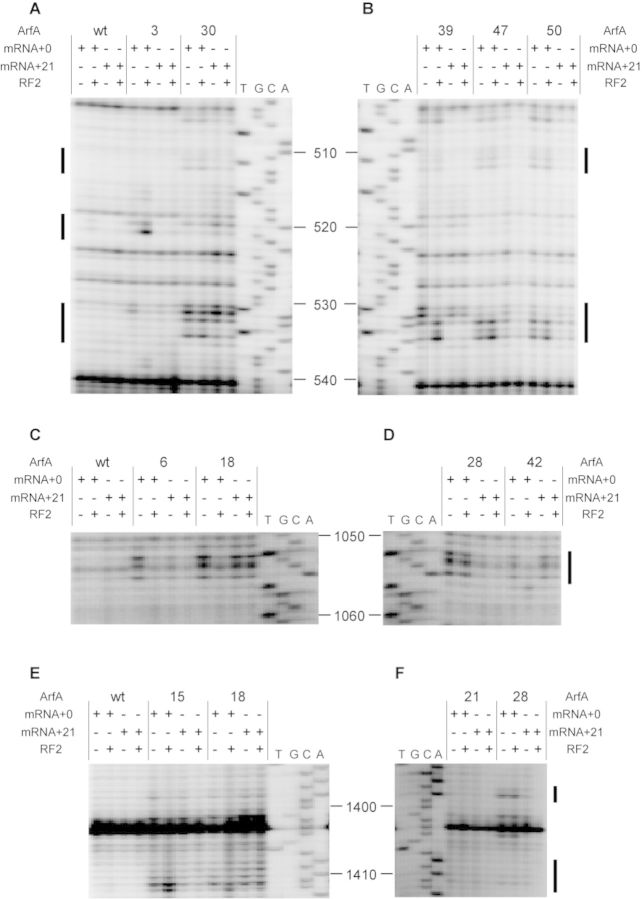
Directed hydroxyl radical probing of 16S rRNA. (**A**–**F**) Stalled ribosome with tRNA^fMet^ occupying the P-site and either mRNA + 0 or mRNA + 21 was probed with ArfA variants in the absence and presence of RF2. The letters A, C, G and T indicate sequencing lanes. The bands of interest are designated by bars.

To test the effect of the length of the 3′ extension of mRNA on cleavage patterns, we selected 11 mutants and performed directed hydroxyl radical probing using mRNA + 21, and the data are compared with those obtained using mRNA + 0 (Figure 6 and Table 1). Some cleavages depended on the length of mRNA in the absence of RF2, i.e. cleavages of 511–513 and 533–535 of 16S rRNA from Fe(II) tethered to positions 47 and 50 of ArfA were detected only when ribosome was stalled on mRNA + 0 (Figure 6A and B). Cleavages in helix 34 (1053–1055) by positions 6 and 28 (Figure 6C and D) and in helix 44 (1398 and 1411–1412) by positions 28 and 15 were detectable only when mRNA + 0 was used (Figure 6E and F). Cleavages of 531–532 from Fe(II) tethered to position 39 were weakened when we used mRNA + 21 instead of mRNA + 0 (Figure 6B). In contrast, residues of 513 and 531–532 from Fe(II) tethered to position 30 were cleaved regardless of the length of mRNA (Figure 6A). Similarly, cleavages in 1053–1055 by Fe(II) tethered to positions 18 and 42 of ArfA did not vary with the length of 3′ extension (Figure 6C and D). Then, we tested the effect of RF2 on cleavage patterns. As shown in Figure 6 and Table 1, some cleavages were affected by RF2 when we used mRNA + 0. In contrast, much fewer changes by RF2 were detected when mRNA + 21 was used.

**Figure 7.**
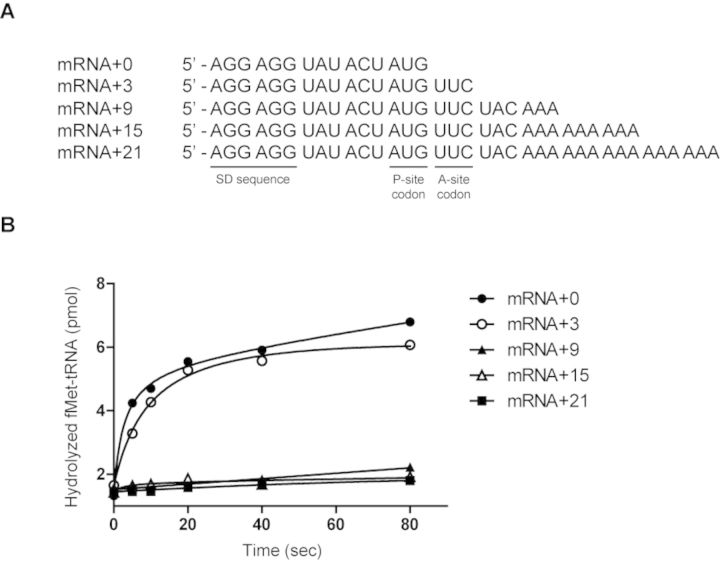
Effect of the length of the 3′ extension of mRNA on PTH activity. (**A**) mRNA sequences used in PTH assay. (**B**) The level of *N*-formyl-[^14^C]Met hydrolyzed from *N*-formyl-[^14^C]Met-tRNA^fMet^ in the P-site of the ribosome stalled on mRNA extended 0, 3, 9, 15 or 21 nucleotides from the P-site codon was measured. Stalled ribosome that contains 70S ribosome, mRNA and *N*-formyl-[^14^C] Met-tRNA^fMet^ was mixed with ArfA and RF2. At specified times, *N*-formyl-[^14^C]Met was extracted by ethyl acetate and radioactivity was measured by a liquid scintillation counter.

We also examined the effect of 3′ extension of mRNA on PTH activity using our *in vitro* assay system, and confirmed that the 3′ extension of mRNA significantly inhibits the hydrolysis of fMet-tRNA^fMet^ (Figure 7A and B). Taken together, these results suggest that RF2 binds to the ribosome stalled on mRNA + 21, although it does not induce conformational change of ArfA required for peptidyl-tRNA hydrolysis.

## DISCUSSION

In the present study, we demonstrated the sites and modes of binding of ArfA to the stalled ribosome. It revealed that the N-terminal region of ArfA is located around the decoding center, while the C-terminal region is located along the mRNA entry channel (Figure 3). After ArfA binding, RF2 enters the stalled ribosome in the absence of stop codon (Figure 5C). Upon RF2 binding, ArfA, including both its N- and C-terminal regions, undergoes a significant conformational change (Figure 3). We also found that RF2 as well as ArfA binds to the ribosome stalled not only in the 3′ end but also in the middle of mRNA (Figure 5B and C), although they can hydrolyze peptidyl-tRNA only in the ribosome stalled at the 3′ end of mRNA. Consistently, productive conformational change of ArfA by RF2 binding occurs only when the ribosome is stalled at the 3′ end of mRNA (Figure 6).

The interaction of the mRNA path with ArfA is reminiscent of that with SmpB during *trans*-translation. The region occupied by the C-terminal half of ArfA essentially overlaps with the region occupied by the C-terminal tail of SmpB (13,18,19). The ArfA-dependent PTH activity decreases with increase of the 3′ extension of mRNA (12) (Figure 7B), and such an inverse correlation has also been found in tmRNA-SmpB-directed *trans*-translation (20,21). These findings raise the possibility that these ribosome rescue factors may recognize the target ribosome through a similar mechanism. Note that ArfA binds to the ribosome regardless of the length of 3′ extension of mRNA (Figure 5A). Recently, we found that tmRNA-SmpB enters the ribosome stalled even in the middle of mRNA to trigger GTP hydrolysis by EF-Tu, whereas subsequent peptidyl-transfer occurs only in the ribosome stalled near the 3′ end of a truncated mRNA (17). Taken together, either ArfA or SmpB initially binds to the region around the decoding center for which the C-terminal region would not compete with the 3′ extension of mRNA. Neither ArfA nor SmpB selects the stalled ribosome free of the 3′ extension of mRNA in the initial step of ribosome rescue. Recently, ArfB (YaeJ), a homolog of class I peptide release factor, has been identified as the third factor that has the capacity for release of the nascent polypeptide from the stalled ribosome (22,23). Interestingly, ArfB (YaeJ) has also the C-terminal tail that occupies the mRNA path in the stalled ribosome (24).

Our finding that ArfA and RF2 bind to the ribosome stalled even in the middle of mRNA is unexpected because the ArfA-dependent peptidyl-tRNA hydrolytic activity by RF2 decreases with an increase of the 3′ extension of mRNA (Figure 7B) and indeed the C-terminal region of ArfA appears to compete with mRNA for the same binding site. Our probing result indicates that ArfA binds to the ribosome stalled on intact mRNA in a different way from that upon binding to the ribosome stalled on a truncated mRNA (Figure 6). Since ArfA and RF2 fail to induce peptidyl-tRNA hydrolysis on the ribosome stalled in a middle of mRNA, the GGQ motif of RF2 would not be accommodated into the peptidyltransferase center. This intermediate complex might possibly reflect ‘the pre-accommodation state,’ which has been believed to occur in the canonical termination process. The crystal structure of ribosome-bound RF2 is considerably different from that of ribosome-unbound RF2 (16,25,26), suggesting that RF2 undergoes significant conformational rearrangement upon recognition of the stop codon in the A-site. Similar conformational rearrangement might occur in the ArfA-dependent ribosome rescue process, i.e. RF2 recognizes the presence of ArfA in the stalled ribosome and undergoes its conformational rearrangement only in the absence of the downstream mRNA to introduce the GGQ motif into the peptidyltransferase center.

**Figure 8.**
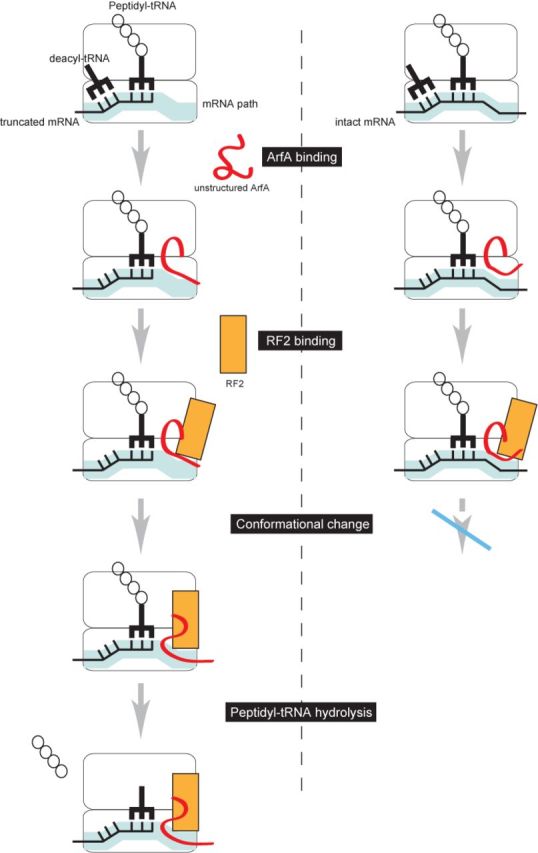
Schematic representation of ArfA/RF2 ribosome rescue. ArfA enters the stalled ribosome regardless of the length of 3′ extension of mRNA. After ArfA binding, RF2 binds to the ribosome, inducing conformational changes of ArfA and RF2 to introduce the C-terminal region of ArfA and the GGQ motif of RF2 into the mRNA entry channel and the peptidyltransferase center, respectively. If the mRNA entry channel is occupied by mRNA, the conformational changes required for peptidyl-tRNA hydrolysis do not occur.

On the basis of the present results, we propose a model of ArfA-dependent ribosome rescue system to explain how ArfA and RF2 recognize the stalled ribosome (Figure 8). Initially, ArfA binds to the stalled ribosome around the decoding center and the mRNA entry channel regardless of the length of the 3′ extension of mRNA, although the binding mode is somewhat different depending on the presence or absence of the 3′ extension. Then, RF2 enters the ArfA-ribosome complex with either no or long 3′ extension, but inducing productive conformational changes of both ArfA and RF2 only in the absence of 3′ extension. Then the C-terminal region of ArfA is fully accommodated into the mRNA entry channel only when the channel is free. Consequently, the C-terminal region may function as the sensor to discriminate between the stalled and translating ribosomes by binding competition between the C-terminal half of ArfA and the 3′ extension of mRNA for the mRNA entry channel.

This model provides several insights into the ArfA/RF2 ribosome rescue system. (i) ArfA binds to the A-site around the decoding center and the mRNA entry channel, (ii) ArfA substitutes for the stop codon to recruit RF2 for ribosome rescue and (iii) the C-terminal region of ArfA functions as the sensor to recognize the mRNA entry channel after RF2 binding.

Unlike in the canonical translational process, RF2 acts on a stalled ribosome as the stop codon-independent peptide release factor in the presence of ArfA. Consistently, RF2 no longer requires the stop codon-recognition motif in the presence of ArfA (6). In case any non-cognate peptidyl-tRNA is in the P-site after misincorporation of non-cognate amino acid into the nascent polypeptide, RF2 can also act as a stop codon-independent peptide release factor, but without ArfA, to interrupt abortive translation (27,28). It poses the fascinating question about whether there is any common underlying mechanism between the two kinds of RF2 actions of the stop codon-independent peptide release with and without ArfA.
